# Renal transplantation during coronavirus pandemic

**DOI:** 10.11604/pamj.supp.2020.35.2.23242

**Published:** 2020-05-04

**Authors:** Nikolaos Garmpis, Christos Damaskos, Anna Garmpi, Alexandros Patsouras, Athanasios Syllaios, Dimitrios Dimitroulis

**Affiliations:** 1 Second Department of Propedeutic Surgery, Laiko General Hospital, Medical School, National and Kapodistrian University of Athens, Athens, Greece; 2 First Department of Propedeutic Internal Medicine, Laiko General Hospital, Medical School, National and Kapodistrian University of Athens, Athens, Greece; 3 Medical School, National and Kapodistrian University of Athens, Athens, Greece; 4 First Department of Surgery, Laiko General Hospital, Medical School, National and Kapodistrian University of Athens, Athens, Greece

**Keywords:** COVID-19, renal, transplantations

## To the editors of the Pan African Medical Journal

Coronavirus disease 2019 (COVID-19) constitutes a new viral infection caused by Severe Acute Respiratory Syndrome Coronavirus 2 (SARS-CoV-2) with worldwide impact [1]. The knowledge concerning its epidemiology and clinical presentation is still evolving. The mortality rate in the general population is estimated at 1-6% [2]. However, patients with chronic renal failure and/or solid organ transplantation are more complicated cases, which cause more diagnostic and therapeutic dilemmas to the physicians, due to comorbidities pre-transplant and immunosuppressive therapies post-transplant. Li et al. demonstrated in a clinical study that COVID-19 can affect the renal function and cause acute renal failure in patients without any renal disfunction in their medical history. The mortality in these patients rises up to 5.3 times, compared to patients without acute renal failure, rendering close monitor of renal function a necessity [3].

Additionally, Guillen et al. reported a case of a kidney transplant recipient, who was infected by SARS-CoV-2. The challenge was both diagnostic and therapeutic. The patient presented with atypical symptoms, mostly gastrointestinal and without respiratory ones initially. After diagnosis, tacrolimus was withdrawn due to the use of Lopinavir/Ritonavir [4]. The patient is still in the ICU with respiratory support, and treatment with both immunosuppression and against SARS-CoV-2. The above information raises various concerns regarding kidney transplantation for both recipients and donors. As far as donors are concerned, they are divided into deceased and living-related. The deceased donor transplants, who had manifested unexplained respiratory failure or had been exposed to confirmed or suspected COVID-19 should be excluded. Should lower or upper airway specimens be obtained in order to test for the virus? Can the CT of thorax be used as a tool for the inclusion or exclusion of the donor?

Is this going to prevent from the virus transmission or will lead to organ wastage? Regarding the transplantation from a living donor, both donors and recipients with symptoms or suspected contacts with COVID-19 are excluded from the transplantation. The procedure can be delayed to a later date, except for the urgent life-saving operations after consent of both donor and recipient. Thus, renal replacement therapy can occur until the existence of safer conditions. The concern is when neither donor and recipient meet any of the above criteria. Does the surgeon operate normally? As Li et al. showed, the infection with SARS-CoV-2 can cause acute renal failure and increase mortality. Is it safe to operate a healthy subject and a patient with chronic renal failure in this contagious environment? Last but not least important, the interaction between the immunosuppressive regimens and the treatment against COVID-19 is not well studied. Can this lead to graft rejection or death due to ineffective treatment of the infection?

## Conclusion

To conclude, COVID-19 created a new reality in all aspects of medicine. New dilemmas and challenges are developing in many aspects of medicine, including renal transplantation. Further studies, reports and approaches are necessary in order to clarify concerns, minimize the risk and improve clinical results.

## Competing interests

The authors declare no competing interests.
